# Antidiarrheal Efficacy and Cellular Mechanisms of a Thai Herbal Remedy

**DOI:** 10.1371/journal.pntd.0002674

**Published:** 2014-02-13

**Authors:** Lukmanee Tradtrantip, Eun-A Ko, Alan S. Verkman

**Affiliations:** Departments of Medicine and Physiology, University of California, San Francisco, San Francisco, California, United States of America; University of Tennessee, United States of America

## Abstract

Screening of herbal remedies for Cl^−^ channel inhibition identified Krisanaklan, a herbal extract used in Thailand for treatment of diarrhea, as an effective antidiarrheal in mouse models of secretory diarrheas with inhibition activity against three Cl^−^ channel targets. Krisanaklan fully inhibited cholera toxin-induced intestinal fluid secretion in a closed-loop mouse model with ∼50% inhibition at a 1∶50 dilution of the extract. Orally administered Krisanaklan (5 µL/g) prevented rotavirus-induced diarrhea in neonatal mice. Short-circuit current measurements showed full inhibition of cAMP and Ca^2+^ agonist-induced Cl^−^ conductance in human colonic epithelial T84 cells, with ∼50% inhibition at a 1∶5,000 dilution of the extract. Krisanaklan also strongly inhibited intestinal smooth muscle contraction in an *ex vivo* preparation. Together with measurements using specific inhibitors, we conclude that the antidiarrheal actions of Krisanaklan include inhibition of luminal CFTR and Ca^2+^-activated Cl^−^ channels in enterocytes. HPLC fractionation indicated that the three Cl^−^ inhibition actions of Krisanaklan are produced by different components in the herbal extract. Testing of individual herbs comprising Krisanaklan indicated that agarwood and clove extracts as primarily responsible for Cl^−^ channel inhibition. The low cost, broad antidiarrheal efficacy, and defined cellular mechanisms of Krisanaklan suggests its potential application for antisecretory therapy of cholera and other enterotoxin-mediated secretory diarrheas in developing countries.

## Author Summary

Secretory diarrhea is a major health challenge in developing countries. Causative agents include bacteria, as in cholera, and viruses, as in childhood rotaviral diarrhea. Though oral rehydration solution (ORS) has reduced mortality from diarrhea four-fold in the last three decades, its efficacy is limited, particularly in the young and elderly, and because of practicalities in its availability and compliance. Antisecretory drug therapy for diarrhea may be efficacious when ORS is not available, as during natural disasters, and it may potentiate the efficacy of ORS. As an alternative approach to the costly and lengthy development of a new chemical entity, in this study we investigated the possibility that effective, natural-product antisecretory therapeutics may already be available, but unappreciated. Screening of diarrhea remedies from around the world for enterocyte chloride channel inhibition identified Krisanaklan, a herbal extract used widely in Thailand for treatment of diarrhea, as effective in inhibiting intestinal chloride secretion. We report the antidiarrheal efficacy and cellular mechanisms of Krisanaklan, providing proof-of-concept for its potential utility for antisecretory therapy of major, life-threatening diarrheas in developing countries.

## Introduction

Secretory diarrhea is a major health challenge in developing countries, representing the second leading cause of mortality globally in children under age 5 [1]. Repeated episodes of hypovolemia from diarrhea can produce malnutrition and impaired development [2]. The mainstay of diarrhea therapy is oral rehydration solution (ORS), which consists of an aqueous mixture of salts and carbohydrates [3], [4]. Though ORS has reduced mortality from diarrhea four-fold in the last 3 decades, its efficacy is limited, particularly in the young and elderly, and because of practicalities in its availability and compliance [5]. Antisecretory drug therapy for diarrhea may be efficacious when ORS is not available, as during natural disasters, and it may potentiate the efficacy of ORS.

The intestinal epithelium absorbs and secretes large volumes of fluid, with net absorption under normal conditions and net secretion in secretory diarrheas. Intestinal fluid secretion involves Cl^−^ transport from the blood into the intestinal lumen through Cl^−^ channels on the enterocyte apical plasma membrane, which include the cAMP-gated channel CFTR (cystic fibrosis transmembrane conductance regulator) and one or more CaCCs (Ca^2+^-activated Cl^−^ channels) whose molecular identity is not known [6]–[8]. CFTR is the primary route for Cl^−^ secretion in secretory diarrheas caused by bacterial enterotoxins in cholera and Travelers' diarrhea (caused by enterotoxigenic E. coli). CaCCs are likely involved as well in these diarrheas because of cross-talk between cyclic nucleotide and Ca^2+^ signaling [9], [10], and may provide the primary route for Cl^−^ secretion in some viral and drug-induced diarrheas, including childhood rotaviral diarrhea [11], [12] and antiretroviral drug-induced diarrhea [13]. The Ca^2+^-activated Cl^−^ channel TMEM16A is expressed intestinal pacemaker cells, the interstitial cells of Cajal, where it is required intestinal smooth muscle contraction and motility [14], [15]. TMEM16A is widely expressed in secretory epithelia in the airways and salivary gland, but probably plays at most a minor role as a CaCC in intestinal epithelium [16].

There is currently no approved antisecretory drug for treatment of major secretory diarrheas such as cholera. Our laboratory has identified, by high-throughput screening, several classes of small-molecule CFTR and CaCC inhibitors (reviewed in ref. [17]), and has shown their efficacy in mouse models of secretory diarrheas [18], [19]. As an alternative approach to the costly and lengthy development of a new chemical entity, here we investigated the possibility that effective, natural-product antisecretory therapeutics may already be available, but unappreciated. Screening of diarrhea remedies from around the world for enterocyte Cl^−^ channel inhibition identified Krisanaklan, a herbal extract used widely in Thailand for treatment of diarrhea, as effective in inhibiting intestinal Cl^−^ secretion and motility. We previously reported that one component of Krisanaklan, eugenol, inhibited the CaCC TMEM16A [20]. Here, we report here on the antidiarrheal efficacy and cellular mechanisms of Krisanaklan, and suggest its potential utility for antisecretory therapy of major, life-threatening diarrheas in developing countries.

## Methods

### Ethics statement

This study was approved by the UCSF Institutional Animal Care and Use Committee (IACUC approved protocol AN089748), and was performed in accordance with the recommendations in the Guide for the Care and Use of Laboratory Animals of the National Institutes of Health.

### Cell lines and herbal preparation

FRT cells stably expressing human CFTR or TMEM16A were generated and cultured as described [16], [21]. T84 cells (ATCC CCL-248) were cultured as described [22]. The Thai herbal formulation Krisanaklan was purchased from Osotspa Inc. (Bangkok, Thailand).

### Short-circuit current

Snapwell inserts containing T84 or FRT cells were mounted in Ussing chambers (Physiologic Instruments, San Diego, CA), as described [16], [23]. Activators and inhibitors were added to the apical solution and an equal volume of vehicle was added at the same time to the basolateral solution. Symmetrical HCO_3_^−^-buffered solutions were used for T84 cells. For FRT cells, the hemichambers were filled with a half-Cl^−^ solution (apical) and the HCO_3_^−^-buffered solution (basolateral), and the basolateral membrane was permeabilized with 250 µg/mL amphotericin B. Under these conditions short-circuit current is a direct measure of apical membrane Cl^−^ conductance. Cells were bathed for a 10 min stabilization period and aerated with 95% O_2_/5% CO_2_ at 37°C. Short-circuit current was measured using an EVC4000 Multi-Channel V/I Clamp (World Precision Instruments, Sarasota, FL).

### Transepithelial transport of Krisanaklan

T84 cells were grown on 12-mm diameter collagen-coated transwell inserts (0.4-µm pore size Costar, Corning, Tewksbury, MA). Cells were cultured for 5–7 days to form tight monolayers with transepithelial resistance 900–1,000 Ω cm^2^. Krisanaklan (1.5 ml of 6% solution) in Ringers bicarbonate buffer was added into the basolateral chamber, and 0.5 ml of Ringers bicarbonate alone was added into the apical chamber. Apical chamber fluid (200 µL) was collected at 0, 30 and 60 min (and replaced with the identical volume of buffer). The fluid samples were bioassayed for Cl^−^ transport inhibition by short-circuit current measurement on T84 cells as described above. The percentage transport of inhibitory substance(s) was computed from activities of apical samples versus the original basolateral fluid, correcting for dilution.

### Fluid secretion and absorption in mouse intestinal closed-loops

Mice (CD1 strain, 25–35 g) were deprived of food for 24 h and anaesthetized with intraperitoneal 2,2,2-tribromoethanol (Avertin, Sigma-Aldrich, St. Louise, MO) (125 mg/kg). Body temperature was maintained at 36–38°C using a heating pad. Following a small abdominal incision, three closed mid-jejunum loops (length 20–30 mm) were isolated by sutures, as described [18]. Loops were injected with 100 µl of PBS or PBS containing cholera toxin (1 µg) without or with Krisanaklan. The abdominal incision was closed with suture and mice were allowed to recover from anesthesia. At 4 h the mice were anaesthetized, intestinal loops were removed, and loop length and weight were measured to quantify net fluid secretion. Fluid absorption was measured separately, from the reduction in loop weight/length ratio at 30 min after injection of 200 µL PBS. PBS containing 10 mM glucose was used as a positive control for fluid absorption. Mice were killed by an overdose of Avertin.

### Gastrointestinal motility

Mice (CD1 strain, weight 25–35 g) were deprived of food for 24 h before experiments. Krisanaklan (3% in 100 µL PBS) was administered either orally or by intraperitoneal injection. Fifteen min later mice were orally administered a charcoal meal (0.2 ml of 10% activated charcoal suspended in 5% gum acacia) with or without 3% Krisanaklan. Thirty minutes later the mice were sacrificed and the small intestine was isolated. The peristaltic index was calculated as the percentage of distance traveled of the charcoal meal relative to the total length of small intestine.

### Rotaviral diarrhea studies

Neonatal C57bl/6 mice (age 5–7 days, weight 1.8–2.5 g) were inoculated with 30 µL (1.2×10^7^ pfu/mL) of Simian SA-11 rotavirus (ATCC VR 1739) by oral gavage, as modified from prior reported models [10], [24]. The treated group received 10 µL Krisanaklan one day after rotavirus infection. Stool specimens were collected by gentle palpation of the mouse abdomen 2 day after rotavirus inoculation. For quantification of stool water content we fabricated a polydimethylsiloxane slab of 1.5-mm thickness with a 1.91-mm diameter hole to contain a cylindrical 4.3-mm^3^ volume of stool, as described [24]. The stool plug was expelled onto absorbent tissue in a humidified atmosphere and allowed to contact the tissue for 1 min. The wetted area was measured and related to absolute water content using stool standards. In some studies the mid-jejunum was perfusion-fixed at 2 days after rotavirus inoculation for preparation of 5-µm thick, hematoxylin and eosin-stained, paraffin-embedded sections.

### Ca^2+^ and cAMP measurement

For measurement of cytosolic Ca^2+^, FRT-TMEM16A cells were plated in 96-well black-walled microplates. After removal of growth medium 100 µl of 10 µM Fluo-4 NW (Invitrogen, Carlsbad, CA) was added and incubated at 37°C for 30 min, then at room temperature for an additional 30 min. Fluo-4 fluorescence was measured with a plate reader at excitation/emission wavelengths of 485/538 nm. cAMP was assayed in T84 cells treated for 30 min with 0 or 10 µM forskolin, without or with Krisanaklan, lysed by repeating freeze/thaw, centrifuged, and the supernatant was assayed (Parameter cAMP immunoassay kit; R&D Systems, Minneapolis, MN).

### High performance liquid chromatography (HLPC) and dialysis

Fractionation was performed on an AKTA Explorer 10 system (GE Healthcare Life Science, Piscataway, NJ) equipped with a C18 reversed-phase column (Varian Pursuit XRs, 250×10 mm, 5 mm particle size, Waldbronn, Germany), as described [20]. In separate studies Krisanaklan was dialyzed using 1-, 10-, and 50- kDa cut-off membranes (Float-A-Lyzer G2, Spectrum Laboratories, Rancho Dominguez, CA).

### Intestinal smooth muscle contraction

Wild-type CD1 mice (age 7–10 weeks) were killed by avertin overdose (200 mg/kg). The ileum was isolated and washed with (in mM): 120 NaCl, 5 KCl, 1 MgCl_2_, 1 CaCl_2_, 10 D-glucose, 5 HEPES, and 25 NaHCO_3_ (pH 7.4). The ends of the ileal segments were tied and connected to a force transducer, as described [25]. Ileal segments were stabilized for 60 min with a resting force of ∼1 mN, with changes of the bathing solution every 20 min.

### Whole-cell patch-clamp

Whole-cell recordings were made at room temperature on T84 cells, and CFTR- and TMEM16A-expressing FRT cells. The bath solution contained (mM): 140 N-methyl-D-glucamine-Cl, 1 CaCl_2_, 1 MgCl_2_, 10 glucose and 10 HEPES (pH 7.4) for the TMEM16A and CFTR. The pipette solution contained (in mM): 130 CsCl, 0.5 EGTA, 1 MgCl_2_, 1 Tris-ATP and 10 HEPES (pH 7.2). TMEM16A was activated by 400 nM free Ca^2+^ in the pipette solution. CFTR currents were recorded by test pulse from −80 to +80 mV from a holding potential of 0 mV in the presence of forskolin. Cl^−^ currents in FRT-TMEM16A cells were elicited by applying voltage pulses from a holding potential of 0 mV to potentials between −100 mV and +100 mV with increases of 20 mV. CaCC was activated by 1000 nM free Ca^2+^ in T84 cells. To record CaCC in T84 cells, external solution contained (mM): 150 NaCl, 6 CsCl, 2 CaCl_2_, 1 MgCl_2_, 10 glucose and 10 HEPES (pH 7.4) were used. The pipette solution contained (in mM): 40 CsCl, 100 Cs-aspartate, 5 EGTA, 1 MgCl_2_, 4.33 CaCl_2_, 4 Na_2_-ATP and 10 HEPES (pH 7.2). The currents in T84 cells were evoked by test pulse from −100 mV to 100 mV with increases of 20 mV from a holding potential of −50 mV. Pipettes (3–4 MΩ) were fabricated on a model P-97 electrode puller (Sutter Instrument, Novato, CA) and polished with a MF-900 Micro Forge (Narishige Scientific Instruments Laboratories). Whole-cell currents were recorded using an Axopatch-200B (Axon Instruments) and currents were filtered at 1–2 kHz and digitized at 2–4 kHz.

### Statistical analysis

Statistical analysis was done with Prism 5 software (GraphPad Software Inc., San Diego, CA) using 2-tailed Student's *t* test, Mann-Whitney rank-sum test, or one-way analysis of variance (ANOVA), where appropriate. Data are presented as the mean ± S.E.M. A *P* value of 0.05 or less was considered significant.

## Results

### A Thai herbal remedy inhibits intestinal cAMP and Ca^2+^-activated Cl^−^ channels

**Figure 1:**
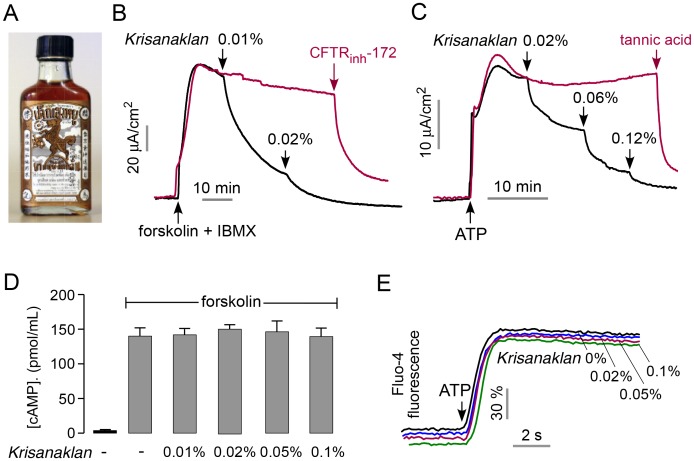
Krisanaklan inhibits intestinal cAMP and Ca^2+^-regulated Cl^−^ channels. A. Krisanaklan, a commonly used Thai herbal antidiarrheal remedy. B. Short-circuit measurement in T84 cells. Representative current trace showing Krisanaklan inhibition of forskolin (20 µM) and IBMX (100 µM)-stimulated CFTR Cl^−^ conductance (black). Parallel study done without added Krisankalan (red) showing inhibition by CFTR_inh_-172. C. Krisanaklan inhibition of ATP (100 µM)-stimulated CaCC Cl^−^ current in T84 cells (black). CFTR was inhibited by pre-addition of CFTR_inh_-172 (20 µM). Parallel study done without added Krisankalan (red), showing inhibition by tannic acid. Data in B and C representative of 4 sets of measurements. D. Cyclic AMP concentration in T84 cell homogenates under basal condition and 30 min after treatment with 20 µM forskolin alone or together with Krisanaklan (mean ± S.E., n = 4). E. Cytoplasmic [Ca^2+^] measured by Fluo-4 fluorescence under basal conditions and following ATP (100 µM). Cells were pretreated with different concentrations of Krisanaklan. Data were slightly displaced in the y-direction to visualize individual curves.

The Thai herbal medicine Krisanaklan (Fig. 1A) was identified from testing of diarrheal remedies for inhibition of intestinal Cl^−^ channels. Fig. 1B shows inhibition of CFTR Cl^−^ current in a human intestinal epithelial cell line (T84 cells) in response to stimulation by the cAMP agonists forskolin, an adenylyl cyclase activator, and IBMX, a phosphodiesterase inhibitor. The IC_50_ for inhibition of CFTR Cl^−^ current was <0.01% Krisanaklan (1∶10,000 dilution), with complete inhibition at higher concentrations. CFTR Cl^−^ current was inhibited by the CFTR inhibitor CFTR_inh_-172 (red curve in Fig. 1B). Krisanaklan also inhibited CaCC Cl^−^ current in T84 cells following stimulation by ATP, with IC_50_ ∼0.02% Krisanaklan (Fig. 1C). The CaCC measurement was done in the presence of a CFTR_inh_-172 to eliminate ATP-dependent CFTR Cl^−^ currents that arise from cross-talk between cAMP and Ca^2+^ signaling. CaCC Cl^−^ current was inhibited by the non-selective CaCC inhibitor tannic acid (red curve in Fig. 1C).

Krisanaklan did not inhibit cAMP or Ca^2+^ signaling in T84 cells. Addition of Krisanaklan up to 0.1% did not reduce cytoplasmic cAMP accumulation in response to forskolin (Fig. 1D), nor did it reduce cytoplasmic Ca^2+^ elevation in response to ATP (Fig. 1E). These results suggest direct action of component(s) of Krisanaklan on CFTR and CaCC Cl^−^ channels.

**Figure 2:**
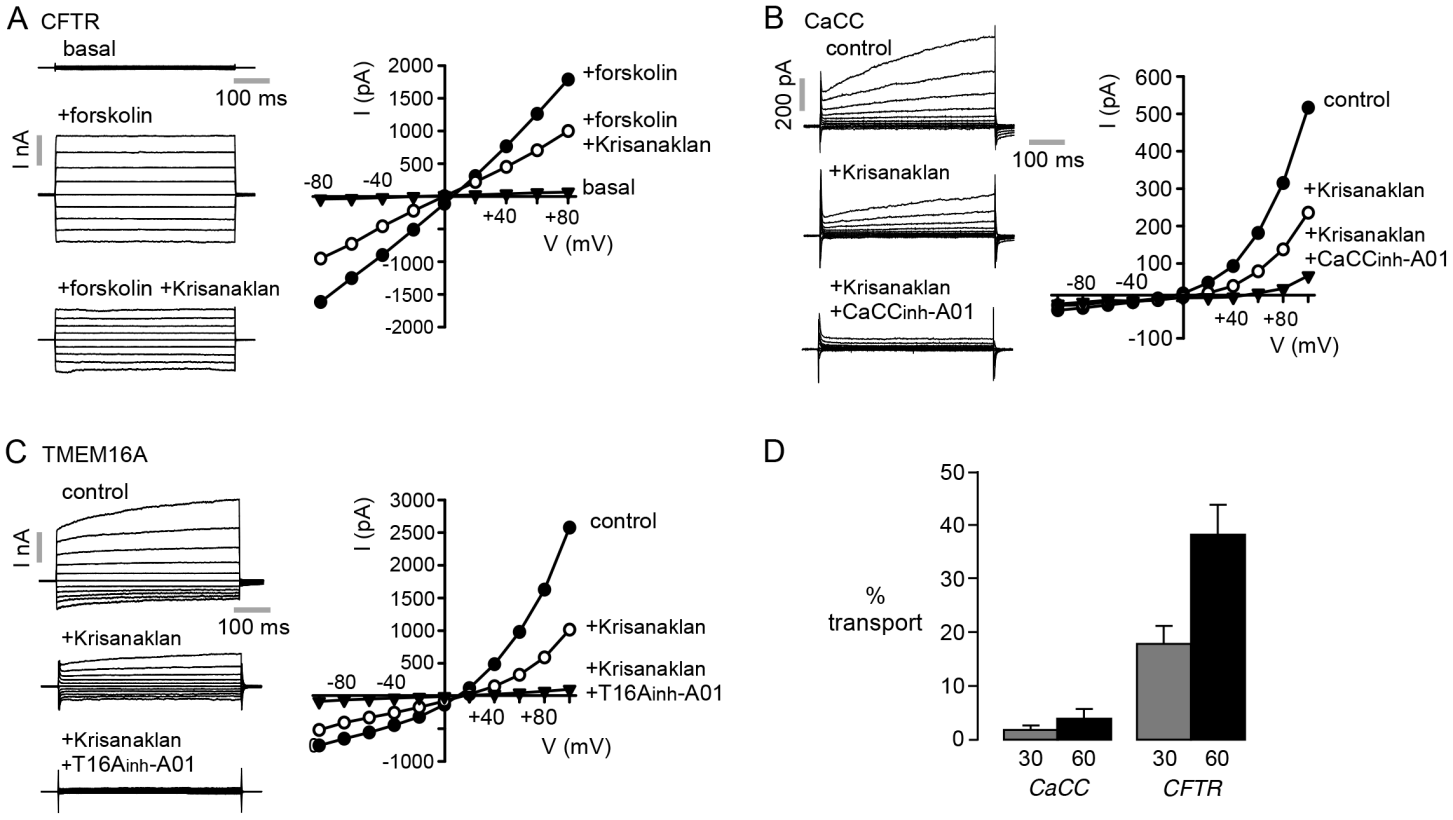
Whole-cell patch-clamp analysis of Krisanaklan Cl^−^ channel inhibition. A. CFTR currents measured at a holding potential of 0−80 mV and +80 mV (in steps of 20 mV) in FRT cells expressing CFTR. Currents were recorded without forskolin (basal), and with 10 µM forskolin without or with Krisanaklan (1∶2000 dilution). (right) Current/voltage (I/V) plot of mean currents at 400 ms. B. (left) CaCC currents recorded in control conditions, in the presence of Krisanaklan, and in the presence of Krisanaklan (1∶2000 dilution) and 100 µM CaCC_inh_-A01. Currents were recorded at voltages of −100 to +100 mV in 20 mV steps from a holding potential of −50 mV. (right) I/V plot of mean currents at 500 ms. C. (left) Whole-cell TMEM16A Cl^−^ currents recorded at a holding potential of 0 mV and pulsing to voltages between −100 and +100 mV (in steps of 20 mV) in TMEM16A-expressing FRT cells. Measurements done in the absence and presence of Krisanaklan (1∶2000 dilution), and TMEM16A inhibitor T16A_inh_-A01 (10 µM). (right) I/V plot of mean currents at 500 ms. D. Transepithelial transport of active components in Krisanaklan in T84 cells grown on a porous transwell insert. Percentage transport of Krisanaklan inhibitory compound(s) at 30 and 60 min measured by bioassay of Cl^−^ channel inhibition (mean ± S.E., n = 3).

Whole-cell patch-clamp was done to further investigate Krisanaklan effects on CFTR and CaCC currents. CFTR Cl^−^ current was measured in CFTR-expressing FRT cells following forskolin addition (Fig. 2A). Approximately linear Cl^−^ currents were seen before and after CFTR inhibition by addition of a 1∶2000 dilution of Krisanaklan. CaCC Cl^−^ current was measured in T84 cells following activation by high pipette Ca^2+^ in the presence of CFTR inhibitor CFTR_inh_-172 (Fig. 2B). Outwardly rectifying Cl^−^ currents were seen before and after Krisanaklan addition, which were fully inhibited by the CaCC inhibitor CaCC_inh_-A01. Cl^−^ current was also measured in FRT cells expressing TMEM16A (Fig. 2C). The outwardly rectifying currents elicited by high pipette Ca^2+^ were ∼50% inhibited by a 1∶2000 dilution of Krisanaklan, and fully inhibited by the TMEM16A inhibitor T16A_inh_-A01.

To investigate whether the active Cl^−^ inhibitory component(s) in Krisanaklan might act from the inside or outside of cells, we used a bioassay to measure transepithelial transport in T84 cells grown on a porous filter. Following addition of Krisanaklan to the basolateral membrane bathing solution, the apical solution was sampled at 30 and 60 min and assayed for CFTR and CaCC activity by short-circuit current in T84 cells. While the component(s) of Krisanaklan responsible for CFTR inhibition were cell permeable, those responsible for CaCC inhibition were not (Fig. 2D). Therefore, different components of Krisanaklan are responsible for CFTR and CaCC inhibition activities, as investigated further below. The results also suggest an intracellular site of action for CFTR inhibition and an extracellular site of action for CaCC inhibition.

### Krisanaklan inhibits intestinal fluid secretion in mouse models of cholera and rotaviral diarrhea

**Figure 3:**
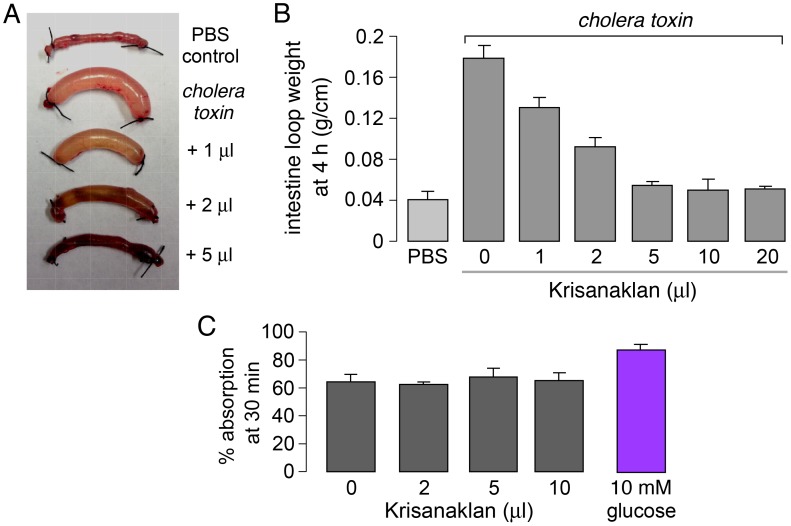
Krisanaklan inhibits fluid secretion in cholera toxin-treated intestinal loops in live mice. A. Closed jejunum loops removed from live mice at 4 µg cholera toxin, without or with Krisanaklan. Administration of 1 µL per loop represents a 1∶100 dilution of Krisanaklan. Saline (PBS) control (no cholera toxin) is shown for comparison (top). B. Intestinal loop weight/length at 4 h (mean ± S.E., 4 mice per group, 14–16 loops studied). C. Krisanaklan does not inhibit intestinal fluid absorption. Intestinal loop were injected with 200 µL buffer alone or with Krisanaklan. PBS containing 10 mM glucose was used as positive control. Loop weight/length was measured at 30 min (mean ± S.E., 4 loops).

Krisanaklan was tested for antisecretory activity in a mouse model of CFTR-dependent secretory diarrhea caused by cholera toxin and of CaCC-dependent secretory diarrhea caused by rotavirus infection. An established model of cholera toxin-induced intestinal fluid secretion was used in which fluid accumulation is measured in closed loops of mouse mid-jejenum *in vivo* at 4 hours after injection of cholera toxin into each loop. Fig. 3A shows marked fluid accumulation in a cholera toxin-injected loop compared to a control (PBS-injected) loop. Inclusion of small quantities of Krisanaklan reduced loop fluid accumulation. Fig. 3B shows a dose-dependent reduction in intestinal fluid accumulation, with IC_50_ of 1–2 µl Krisanaklan per loop, with near complete inhibition of loop fluid accumulation at higher concentrations.

The determinants of intestinal fluid accumulation include fluid secretion and absorption. To verify that Krisanaklan did not affect intestinal fluid absorption, measurements of fluid absorption were made in closed, mid-jejunal loops at 30 min after injection of 200 µl PBS, in which ∼65% of the injected fluid was absorbed. Fig. 3C shows no significant effects of Krisanaklan on loop fluid absorption.

**Figure 4:**
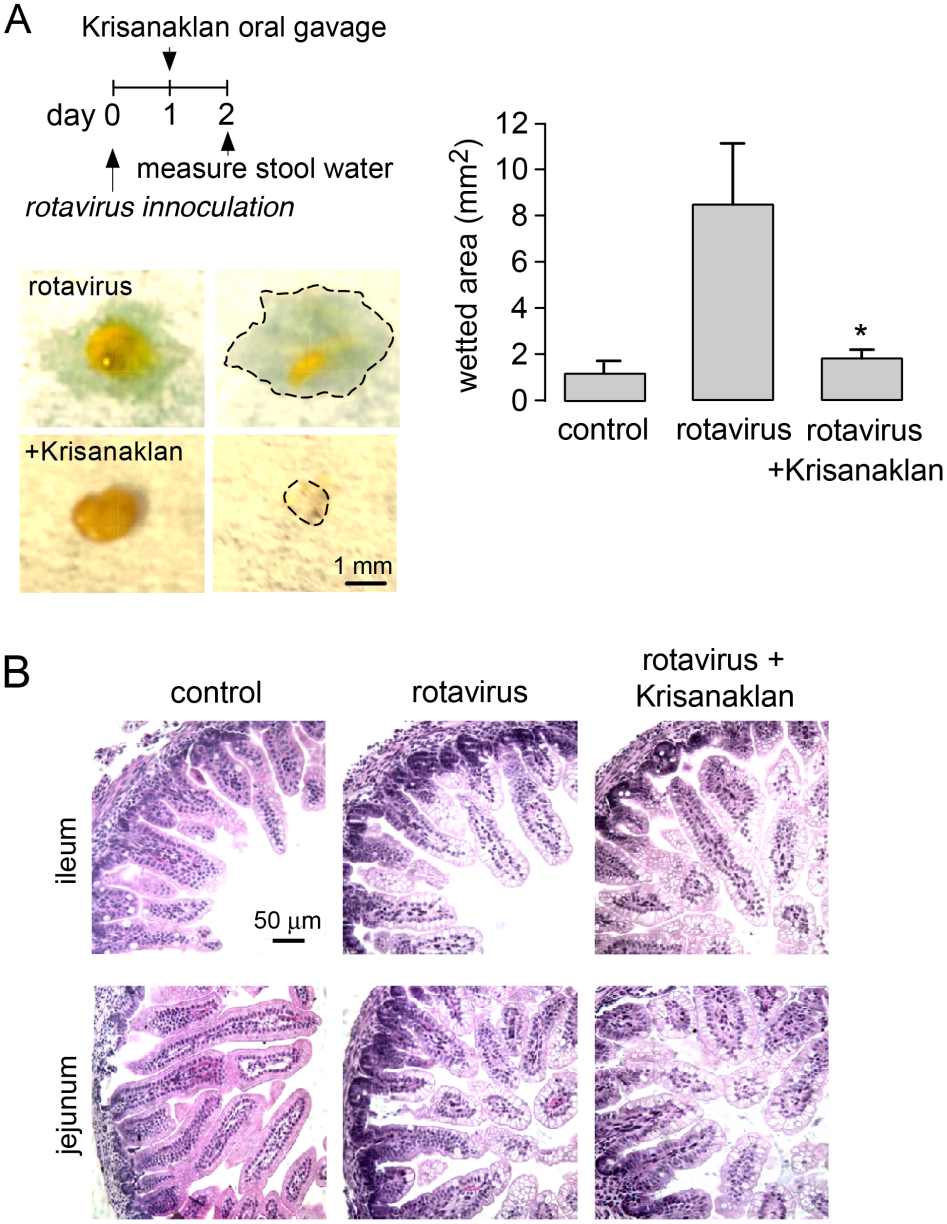
Krisanaklan prevents watery diarrhea in rotavirus-inoculated neonatal mice. A. (left) Neonatal mice were inoculated with rotavirus by oral gavage, followed by Krisanaklan (or saline control) at day 1, and stool water was determined at day 2. Photographs of stool obtained from rotavirus-inoculated mice without and with Krisanaklan treatment. Stool was contacted with absorbent paper for 1 min to allow wetting (demarcated by dashed line). (right) Stool water content deduced from the wetted area on absorbent paper following deposition of a defined stool volume (mean ± S.E., 8 mice per group, * P<0.005). B. Hematoxylin and eosin-stained sections of ileum and jejunum from control mice, and untreated and Krisanaklan-treated rotavirus-inoculated mice.

Rotaviral diarrhea in neonates is thought to result from activation of CaCC by the rotaviral enterotoxin NSP4, which causes elevation of cytoplasmic Ca^2+^ in enterocytes by mechanisms involving enteric nerves, and perhaps galanin or integrin receptors [26]–[28]. To study Krisanaklan action, neonatal mice were inoculated with live rotavirus by oral gavage, which consistently produced watery diarrhea 1–3 days later. A single dose of Krisanaklan (or saline control) was administered at day 1, and stool water content was determined at day 2. Fig. 4A (left) shows watery stool in rotavirus-inoculated mice, and near-normal, non-watery stool in the Krisanaklan-treated mice. Stool water content was judged both from stool appearance, and semi-quantitatively from the wetted area on absorbent paper after deposition of a defined stool volume (Fig. 4A, right).

The prevention of watery stool by Krisanaklan could be a result of its antisecretory action and/or inhibition of rotaviral infection of the intestine. Fig. 4B shows the most characteristic finding of rotaviral infection of the small intestine, prominent enterocyte vacuolization [29]. Similar pathological changes were seen in intestine from Krisanaklan-treated mice, suggesting that Krisanaklan did not prevent the rotavirus infection.

### Krisanaklan inhibits intestinal smooth muscle contraction

**Figure 5:**
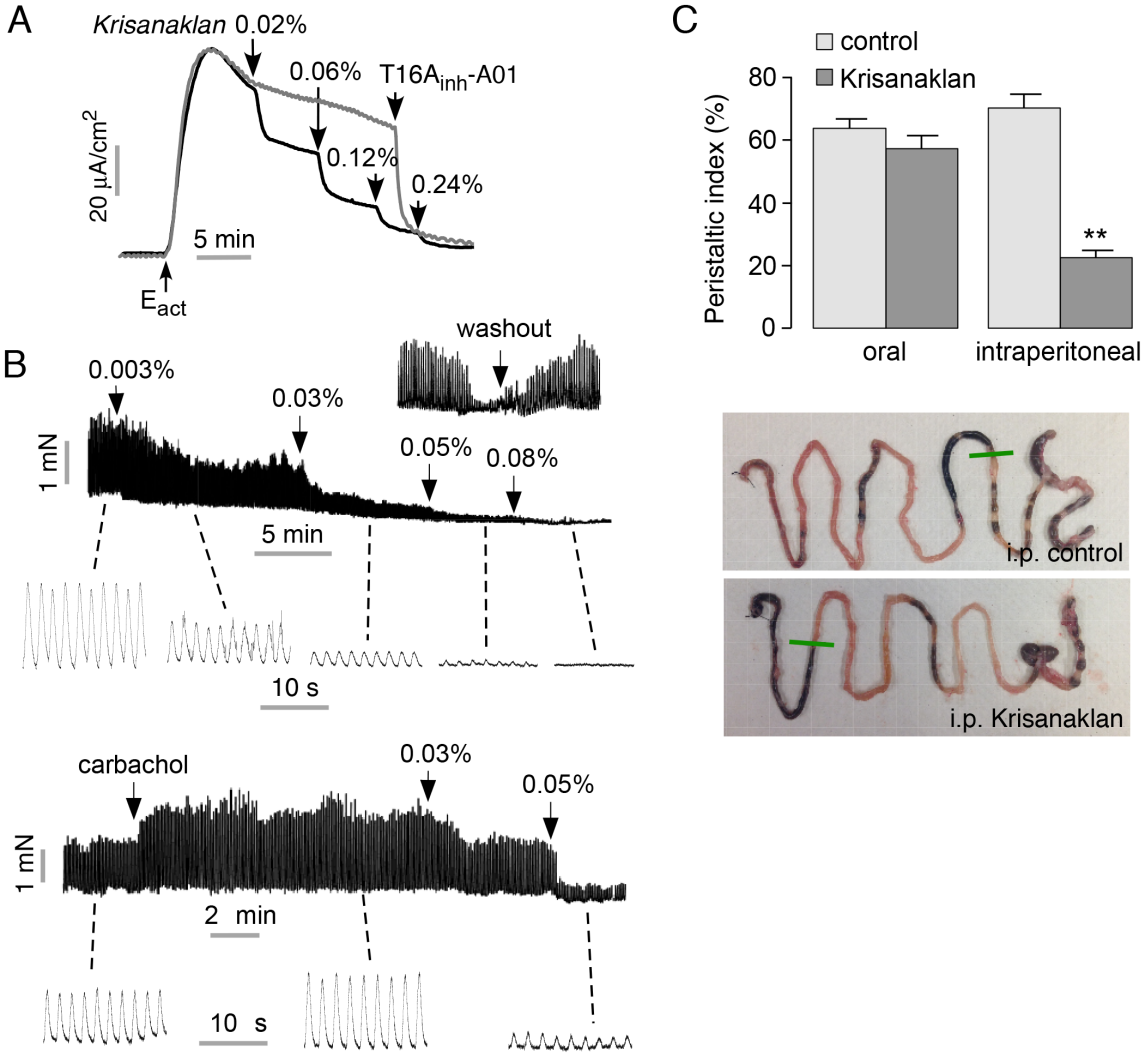
Krisanaklan inhibits TMEM16A Cl^−^ current and intestinal contraction. A. Short-circuit current in TMEM16A-expressing FRT cells, showing Krisanaklan inhibition of 10 µM E_act_ (a TMEM16A activator)-stimulated TMEM16A Cl^−^ current. Measurements were made following permeabilization of the basolateral membrane and in the presence of a transepithelial Cl^−^ gradient (see Methods). Parallel study done with added Krisanaklan (grey curve); where indicated 5 µM T16A_inh_-A01 (a TMEM16A inhibitor) was added. B. (top) Contractile force generated spontaneously by mouse ileal segment showing inhibition by Krisanaklan. (bottom) Krisanaklan inhibition of ileal contraction after stimulation by carbachol. Contraction data are representative of 3 sets of experiments. (inset) Reversibility of Krisanaklan action following washout. C. (top) Peristaltic index in mice receiving 3% Krisanaklan by intraperitoneal injection or oral gavage, compared to water control (mean ± S.E., 4 mice per group, ** P<0.01). (bottom) Representative photographs of small intestine, showing distance traveled of an activated charcoal meal.

Based on our prior study of TMEM16A inhibition by Krisanaklan [20], we postulated that the antidiarrheal action Krisanaklan may also involve a third mechanism – inhibition of intestinal smooth muscle contraction, as TMEM16A is expressed in interstitial cells of Cajal, where it is required for intestinal smooth muscle contraction [14]. Fig. 5A shows Krisanaklan inhibition of TMEM16A Cl^−^ current in TMEM16A-expressing FRT cells, with IC_50_ ∼0.06% Krisanaklan, and complete inhibition at higher concentrations.

Krisanaklan inhibition of intestinal smooth muscle contraction was measured in *ex vivo* mouse ileal strips using a force transducer and a 37°C physiological bath. Fig. 5B (top) shows spontaneous ileal contractions with amplitude ∼1.5 mN. In agreement with our prior data [20], addition of Krisanaklan to the bath produced a concentration-dependent reduction, to near zero, of contraction amplitude, without effect on contraction frequency. Krisanaklan also reduced the amplitude of intestinal contractions following application of the agonist carbachol (Fig. 5B, bottom).

To investigate whether Krisanaklan inhibition of intestinal smooth muscle contraction found *ex vivo* may be relevant to gastrointestinal motility *in vivo*, we used a standard assay of intestinal motility involving transit of an orally administered activated charcoal meal. While intraperitoneal Krisanaklan at a dose similar to that used in humans significantly reduced peristaltic index, oral Krisanaklan did not (Fig. 5C). The difference is likely due to minimal accumulation of TMEM16A-inhibiting components in Krisanaklan in interstitial cells of Cajal in the intestinal wall following oral administration.

### Distinct components of Krisanaklan are responsible for Cl^−^ channel inhibition

**Figure 6:**
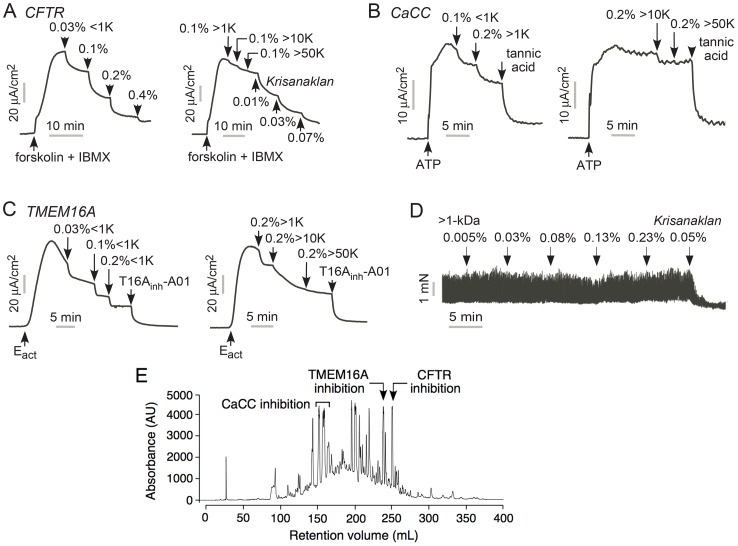
Distinct compounds are responsible for the Cl^−^ channel inhibition activities of Krisanaklan. A. Short-circuit current in T84 cells showing inhibition of (A) CFTR, (B) CaCC, and (C) TMEM16A Cl^−^ current by size fractions of Krisanaklan prepared by dialysis (using 1-, 10- and 50-kDa cut-off membranes). In A, different concentrations of the <1-kDa fraction tested (left) and of the >50-KDa fractions tested following single additions of the >1-kDa and >10-kDa fractions (right). Data in A-C representative of 4 sets of experiments. D. Contractile force in ileal strips (as in Fig. 4B), showing lack of effect of >1-kDa dialysis fraction. E. HPLC fractionation showing chromatogram at 280 nm absorbance, adapted from prior work [20]. Fractions with CFTR, CaCC and TMEM16A activities indicated.

We investigated the nature of the component(s) responsible for Cl^−^ channel inhibition by Krisanaklan. Initial studies showed that the Cl^−^ channel inhibition activities of Krisanaklan were heat-insensitive (100°C for 2 min, data not shown). Several rough size fractions of Krisanaklan were prepared by dialysis using 1-, 10- and 50-kDa cut-off membranes and tested for Cl^−^ channel inhibition. Fig. 6A shows inhibition of CFTR by the <1 kDa fraction, but little effect of the >1, >10 and >50 kDa size fractions, suggesting that the CFTR inhibitor molecule(s) have molecular size <1 kDa. Similar CaCC inhibition was seen for <1 and >1 kDa size fractions, whereas the >10 and >50 kDa showed little inhibition (Fig. 6B). Strong TMEM16A inhibition was seen for the <1 kDa fraction, with less inhibition for the higher molecular size fractions (Fig. 6C), suggesting that the TMEM16A inhibitor molecule(s) have a molecular size <1 kDa. Fig. 6D shows that the >1 kDa fraction produce little inhibition of intestinal smooth muscle contraction, whereas the original Krisanaklan showed strong inhibition. Fig. 6E shows reverse-phase HPLC fractionation of Krisanaklan, done as reported previously [20]. Testing of individual fractions reveals distinct fractions as responsible for the CFTR, CaCC and TMEM16A inhibition actions of Krisanaklan. CaCC inhibition activity was found in several fractions, suggest a heterogeneous mixture of relatively large molecules as responsible.

**Figure 7:**
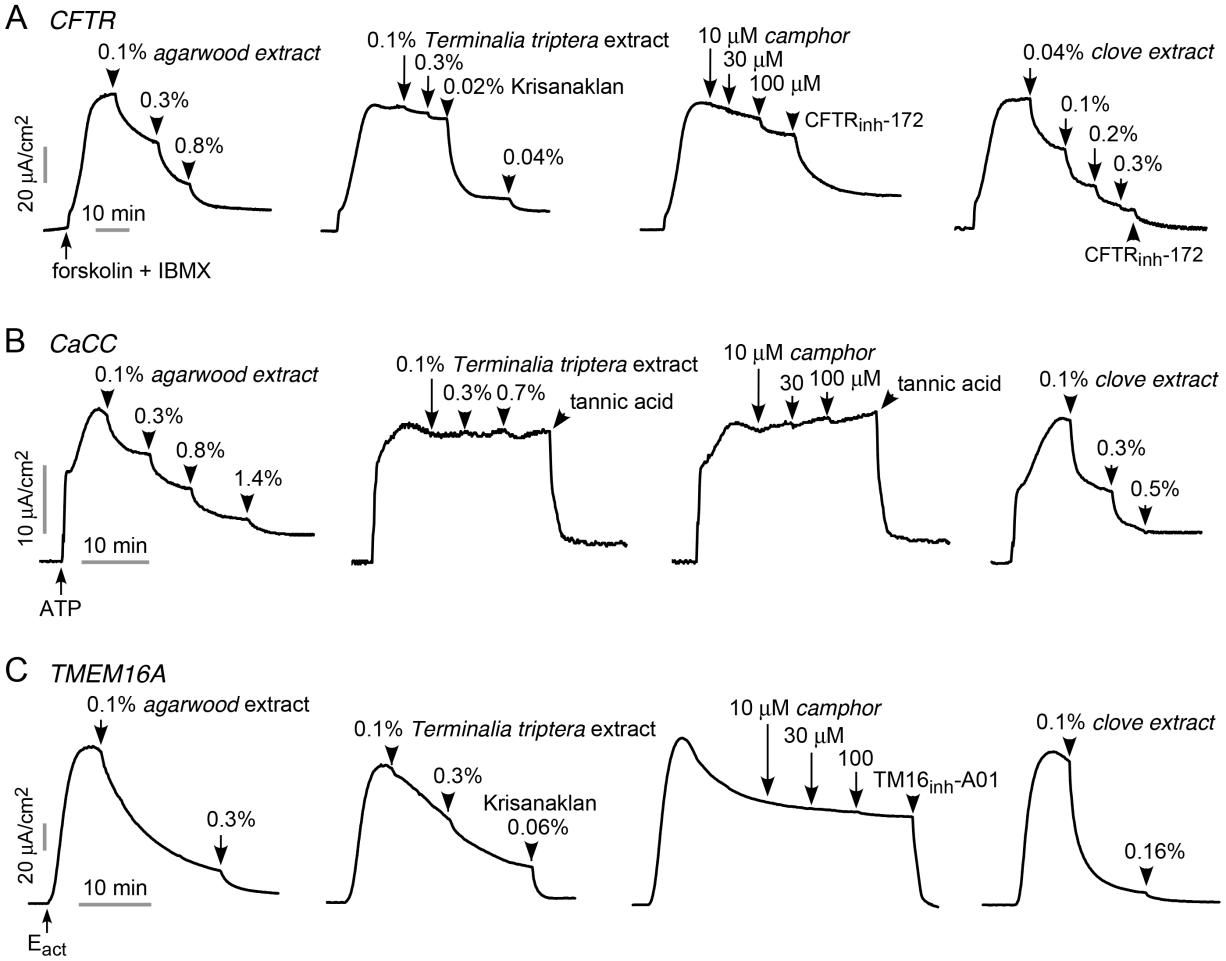
Herbal constituents responsible for the Cl^−^ channel inhibition activities of Krisanaklan. A. Short-circuit current in T84 cells showing inhibition of forskolin (20 µM) and IBMX (100 µM)-stimulated CFTR Cl^−^ current by extracts from agarwood, *Terminalia triptera*, camphor and clove. B. Short-circuit current in T84 cells showing inhibition of ATP (100 µM)-stimulated CaCC Cl^−^ current by extracts. C. Short-circuit current in FRT-TMEM16A cells showing inhibition of E_act_ (10 µM)-stimulated TMEM16A Cl^−^ current by extracts. Data representative of 3 sets of experiments.

To determine which of the four herbal constituents of Krisanaklan are responsible for its Cl^−^ channel inhibition activities, extracts were prepared from each individual herb and tested in T84 and FRT-TMEM16A cell cultures. Concentrations were adjusted to correspond to the original Krisanaklan formulation consisting of an ethanol/water (54∶46) extract in which each 100 mL is extracted from 10 g *Aquilaria crassna* bark (agarwood), 33.3 g clove flower bud, 2 g *Terminalia triptera Stapf* bark and 4.8 g camphor. CFTR inhibition activity was found in the agarwood and clove tracts, but not in the camphor and *Terminalia triptera* extracts (Fig. 7A). CaCC inhibition activity was found in the agarwood and clove extracts, but not in the camphor and *Terminalia triptera* extracts (Fig. 7B). TMEM16A inhibition activity was found mainly in the agarwood and clove extracts (Fig. 7C).

## Discussion

There is an unmet need for effective drug therapy for secretory diarrheas, especially in developing countries where cholera and other enterotoxin-mediated secretory diarrheas remain a major cause of morbidity and mortality. Potential targets for antisecretory therapy include the causative bacterial or viral agent (vaccines and antibiotics), elaborated endotoxins and endotoxin-enterocyte interactions, as well as enterocyte signaling effectors (cAMP, cGMP, Ca^2+^) and membrane transporters involved in fluid secretion (Cl^−^ and K^+^ channels, NKCC1) and absorption (NHE3, SGLT1) [6]. Cl^−^ channels are attractive targets for antisecretory therapy because they are the final, rate-limiting step in Cl^−^ (and hence Na^+^ and water) secretion. Unlike vaccines and antimicrobials that target the causative microbial agent, therapies targeting host secretory mechanisms, such as enterocyte Cl^−^ channels, are not subject to the emergence of resistance. Here, we identified a widely used Thai herbal remedy, Krisanaklan, as having broad antidiarrheal efficacy in bacterial and viral models of secretory diarrhea, which, at the cellular level, inhibits the two major enterocyte Cl^−^ channels, CFTR and CaCC.

CFTR and CaCCs are responsible for Cl^−^ secretion across the luminal membrane of enterocytes in the intestinal epithelium. Several lines of evidence support the conclusion that CFTR is the major apical membrane Cl^−^ pathway in secretory diarrheas caused by the bacterial enterotoxins in cholera and Traveler's diarrhea; *(i)* The small intestine and colon show robust cAMP-activated CFTR Cl^−^ currents [30]; *(ii)* intestinal Cl^−^ and fluid secretion are reduced in CFTR-deficient mice and humans [31]–[33]; and *(iii)* CFTR inhibitors are effective in various rodent models of cholera [18], [19]. CaCC(s) are likely involved as well in diarrheas caused by bacterial endotoxins, as experimental evidence supports cross-talk in cAMP and signalling mechanisms in which cAMP elevation increases cytoplasmic Ca^+2^ [9] and Ca^+2^ elevation increases cytoplasmic cAMP [34]. CaCC(s) are proposed to be the primary route for Cl^−^ secretion in diarrheas caused by rotaviral and other viral enterotoxins [24], [35] and various anti-retroviral and chemotherapeutic agents [13], [36]; however, definitive quantification of the involvement of CaCC(s) in diarrheas awaits their molecular identification. From these considerations therapeutics targeting both enterocyte CFTR and CaCC(s) are predicted to have the greatest and broadest efficacy in secretory diarrheas.

Krisanaklan is an inexpensive, natural-product extract containing ingredients that fully inhibit the major enterocyte Cl^−^ channels, CFTR and CaCC. There are two antisecretory agents currently under clinical evaluation, one natural product and one synthetic small molecule. Crofelemer, a mixture of proanthocyanidin oligomers extracted from the bark latex of *Croton lechleri*, was recently approved for HIV-associated diarrhea [37]. Crofelemer is a weak and partial inhibitor of CFTR (IC_50_>100 µM), though it fully inhibits enterocyte CaCC, albeit with low potency (IC_50_∼10 µM) [23]. Crofelemer is thus unlikely to be beneficial in secretory diarrheas such as cholera and Traveler's diarrhea in which CFTR is the major Cl^−^ secretory pathway and in which fluid secretion is very high. A small molecule, iOWH032, is in clinical trials for cholera [38]. iOWH032 is a close chemical analog of the glycine hydrazide GlyH-101 [39] that targets the extracellular (lumen-facing) surface of CFTR. However, iOWH032 has low CFTR inhibition potency (IC_50_>5 µM) and hence rapid (seconds or less) dissociation from CFTR. Mathematical modeling of an orally administered drug targeting the extracellular surface of intestinal crypts predicts little antisecretory efficacy of a micromolar-affinity CFTR inhibitor under conditions of high fluid secretion because of convective washout [40]. Alternative candidates for CFTR-targeted antidiarrheal therapy include glycine hydrazide conjugates with IC_50_∼50 nM that resist convective washout [19], [41], and thiazolidinones and quinoxalinediones that act on the cytoplasmic surface of CFTR with IC_50_ as low as 4 nM [18], [21], [42], [43].

The three distinct actions of Krisanaklan, including inhibition of CFTR and non-TMEM16A CaCC(s), and TMEM16A, are mediated by different components of the herbal extract. HLPC fractionation showed each of the inhibition activities in different fractions, and testing of size fractions prepared by dialysis indicated that small molecules of <1 kDa molecular size account for the CFTR and TMEM16A inhibition activities, and more heterogeneous, larger molecules for CaCC inhibition. We previously reported that the small molecule eugenol, a major component of clove, as a small-molecule TMEM16A inhibitor that likely accounts, at least in part, for the TMEM16A inhibition activity of Krisanaklan [20]. The molecular identities of the CFTR and CaCC inhibitors in Krisanaklan were not determined in this study, though testing of individual herbs suggest that they arise from two of the four herbal constituents, agarwood and clove. Based on prior studies of Crofelemer [23] and red wines [44], the compounds responsible for CaCC inhibition are probably relatively large, heterogeneous and polyphenolic, whose molecular identities would be very difficult to determine. Agarwood extracts have been shown to contain several classes of phytochemical components including alkaloids, saponin, tannins, anthroquinones, glycosides and triterpenoids [45], [46], some of which may be responsible its Cl^−^ channel inhibition activity. Clove is the dried flower bud of *Caryephyllus aromaticus L*, which contains the volatile compound eugenol, as well as non-volatile tannins, flavonoids, sterols and glycosides [47], [48]. Though eugenol and tannins lack CFTR inhibition activity [20], [44], flavonoids are known to bind to CFTR and may be responsible for CFTR inhibition.

Our results suggest that Krisanaklan, or extracts/components from its individual herbal constituents, is a potential candidate for antisecretory therapy of life-threatening diarrheas in developing countries. The potential advantages of Krisanaklan over alternative antisecretory agents under development include broad Cl^−^ channel specificity with proven efficacy in mouse models, a long history of use in adults and children, low cost, and immediate availability for clinical testing. However, data from *in vitro* and animal models should be extrapolated cautiously to human diarrheas because of differences in intestinal anatomy, fluid secretion rates and, potentially, enterocyte signaling mechanisms. We also note that, as found for vaccines, the efficacy of antisecretory therapeutics may differ in different target populations because of genetic and environment factors. Notwithstanding these caveats, the preclinical data reported here support clinical trials of Krisanaklan for antisecretory therapy of diarrheas.
